# Recovering from Intimate Partner Violence through Strengths and Empowerment (RISE): Initial Evaluation of the Clinical Effects of RISE Administered in Routine Care in the US Veterans Health Administration

**DOI:** 10.3390/ijerph19148793

**Published:** 2022-07-20

**Authors:** Katherine M. Iverson, Sara B. Danitz, Stephanie K. Low, Jennifer A. Knetig, Kathryn W. Doyle, LeAnn E. Bruce

**Affiliations:** 1 Women’s Health Sciences Division of the National Center for PTSD, VA Boston Healthcare System, Boston, MA 02130, USA; sara.danitz@va.gov (S.B.D.); stephanie.low@va.gov (S.K.L.); 2 Department of Psychiatry, Boston University School of Medicine, Boston, MA 02118, USA; 3 VA Northeast Ohio Healthcare System, Cleveland, OH 44106, USA; jennifer.knetig@va.gov; 4 VA Phoenix Healthcare System, Phoenix, AZ 85012, USA; kathryn.doyle@va.gov; 5 Intimate Partner Violence Assistance Program, Care Management and Social Work, Department of Veterans Affairs, Washington, DC 20420, USA; leann.bruce@va.gov; 6 Department of Social Work, Western Kentucky University, Bowling Green, KY 42101, USA

**Keywords:** Intimate Partner Violence Assistance Program (IPVAP), self-efficacy, trauma, treatment, Veterans

## Abstract

Intimate partner violence (IPV) is a common concern among military Veterans that negatively impacts health. The United States’ Veterans Health Administration (VHA) has launched a national IPV Assistance Program (IPVAP) to provide comprehensive services to Veterans, their families and caregivers, and VHA employees who use or experience IPV. Grounded in a holistic, Veteran-centered psychosocial rehabilitation framework that guides all facets of the program, the IPVAP initiated the pilot implementation of a novel intervention called Recovering from IPV through Strengths and Empowerment (RISE). This evidence-based, person-centered, trauma-informed, and empowerment-oriented brief counseling intervention is designed to support those who experience IPV and to improve their psychosocial wellbeing. This program evaluation study describes clinical outcomes from patients who participated in a pilot implementation of RISE in routine care. We examined changes in general self-efficacy, depression, and valued living, as well as treatment satisfaction among patients who received RISE and completed program evaluation measures at VHA facilities during the pilot. Results from 45 patients (84% women) indicate that RISE was associated with significant pretreatment to posttreatment improvements in self-efficacy, depression, and valued living (Cohen’s *d* s of 0.97, 1.09, and 0.51, respectively). Patients reported high satisfaction with treatment. Though preliminary results were similar across gender and IPV types, findings from the evaluation of the pilot implementation of RISE demonstrate the intervention’s feasibility, acceptability, and clinical utility in routine VHA care and inform the scalability of RISE. Additionally, findings provide preliminary support for the effectiveness and acceptability of RISE with men. Modification to RISE and its implementation are discussed, which may be useful to other settings implementing IPV interventions.

## 1. Introduction

Intimate partner violence (IPV) is a public health crisis that has catastrophic rippling effects on individuals, families, and communities [1]. IPV experiences include any physical or sexual violence, stalking, or psychological aggression (including coercive acts and controlling behavior) by a current or former intimate partner. Worldwide, an estimated 27% of women and girls aged 15 and older have experienced physical and sexual IPV [2], but there are also high rates of IPV experienced by other groups, including men and gender and sexual minorities [3,4,5]. IPV is a significant concern among individuals who have served in the military, particularly the Veteran population [6,7]. In the United States (US), women Veterans are 1.6 times more likely to experience IPV during their lifetime than women who never served in the military [8]. A recent review found that more than one in four women Veterans (26%) experienced IPV in the past-year [9]. There is relatively limited data on IPV experiences among Veteran men [9] and much of the research to date has focused on their use (perpetration) of violence [10,11]; however, several studies suggest IPV experiences are a significant issue impacting their health and functioning [12,13,14].

IPV is a common social determinant of health that can cause and exacerbate physical, mental, and social health issues [4,15,16,17]. It is well-documented that Veterans who experience IPV often suffer substantial health impacts, which may be compounded by prior stressors during and following military service [18,19]. For example, experiences of IPV are strongly associated with posttraumatic stress disorder (PTSD), substance use disorders, depression, anxiety, and somatic symptoms, as well as work-related impairment among male and female Veterans after adjusting for military sexual trauma and/or combat experiences [13,20,21,22]. With Veterans making up 10% of the US population, addressing IPV experiences among this population is critical [6].

In the US, following recommendations from a national Domestic Violence/IPV Task Force [23], the Veterans Health Administration (VHA) developed a national IPV Assistance Program (IPVAP) whose mission is to implement comprehensive Veteran-centered, recovery-oriented services for Veterans, their families and caregivers, and VHA employees who use or experience IPV [24]. These services are ground in a holistic, trauma-informed psychosocial rehabilitation framework that guides all aspects of the IPVAP. A critical component of the IPVAP is ensuring that all VA Medical Centers have IPVAP Coordinators (typically social workers or psychologists) who are responsible for raising awareness, building community partnerships, and integrating clinical initiatives into care for Veterans who use or experience IPV. For example, IPVAP Coordinators have played a critical role in supporting the implementation of Strength at Home, a trauma-informed evidence-based group treatment for Veterans who use IPV [25,26].

Clinical initiatives to assist Veterans who experience IPV include the integration of trauma-informed IPV screening practices to identify Veterans who experience IPV. As per VHA guidance [23,24], patients who disclose IPV are offered supportive education, resources, and psychosocial service referrals. Although IPV screening practices in VHA have increased over the years, particularly for women [27,28], few VHA facilities offer treatments for Veterans experiencing IPV beyond advocacy-based consultative services and safety planning. Moreover, although evidence-based psychotherapies are available through mental health, most of these interventions have not been validated for addressing ongoing IPV and not all individuals who experience IPV have a mental health condition or want intensive mental health treatment. Further, there is variability in the evidence for such services in addressing current IPV due to variability of intervention types and settings, limiting our understanding of how to best deliver IPV care [29]. Thus, although VHA’s existing advocacy-based consultation services and evidence-based psychotherapies are important components of a comprehensive healthcare response to IPV, there are gaps in VHA care in terms of manualized IPV-specific psychosocial counseling interventions to support Veterans who experience IPV. Specifically, there is a need for flexible and individualized counseling interventions that are a middle-ground between consultation and more intensive psychotherapy for mental health conditions.

Recovering from IPV through Strengths and Empowerment (RISE) was developed to fill this gap in VHA care. RISE is a brief, transdiagnostic, Motivational Interviewing (MI)-based psychosocial counseling intervention [30] that is grounded in principles of empowerment and trauma-informed care [31,32,33,34]. RISE embodies the key tenants of trauma-informed and empowerment-oriented care through its focus on understanding patients’ values and aiming to facilitate, rather than direct, change in the service of empowering patients to have voice and choice in their care and treatment goals [34,35]. RISE targets general self-efficacy and personal empowerment, as they are critical factors that increase mental health, quality of life, and safety over time [36,37,38,39,40]. Accordingly, both the advocacy and psychological trauma literature recognize increased empowerment and self-efficacy as important mechanisms of healing and recovery from IPV [31,32,35,41,42,43,44,45,46].

RISE is delivered individually and was designed to be both variable-length and modular to maximize flexibility, choice, and autonomy, as well as to better meet both clinician and patient needs, preferences, values, and circumstances. Patients choose which modules to focus on during each session from a menu of six options. The original RISE intervention that was piloted in this manuscript included up to six sessions (RISE was initially designed to be up to 6 sessions [30], but it has since been expanded to be up to 8 sessions, to maximize flexibility and skills generalization) and included six modules that include Safety Planning, Education on Health Effects of IPV and Warning Signs, Improving Coping and Self-Care, Enhancing Social Support, Making Difficult Decisions, and Connecting with Resources and Moving Forward [30,47]. Table 1 provides brief descriptions of each of these modules, which were developed based on the IPV literature and formative research on Veterans’ preferences for IPV counseling [48,49]. RISE also incorporates elements of MI [50], an evidence-based approach to behavioral health change that involves recognition that motivation to change is elicited from the patient (not imposed by others), and involves navigating ambivalence, rolling with resistance, and personalized goal setting to facilitate changes that are consistent with patients’ values (i.e., valued living). MI-based approaches have support for their acceptability and clinical utility for addressing IPV and other forms of interpersonal violence [45,51,52,53]. MI emphasizes self-efficacy and empowerment using a collaborative approach focusing on changes that individuals can safely control by themselves even in the context of an abusive relationship, particularly in the domains addressed in the modules (e.g., enhancing knowledge of IPV health effects and warning signs, increasing self-care, and coping in everyday life; Table 1).

In the first session, patients partake in an orientation to RISE and its overall structure, and then they are invited to share their experiences with IPV and their associated goals for treatment. Next, the clinician introduces the concepts of general self-efficacy and elicits patients’ perspectives on their own sense of self-efficacy. Patients receive a ‘menu of options’ handout that describes each module and select the module they would like to focus on that session, based on their current needs and preferences. Modules can be selected in any order and patients do not need to complete all the modules, and they can repeat modules if desired. Each module includes experiential exercises and worksheets to facilitate knowledge and skill acquisition and personalized goal setting. Patients are invited to set behaviorally specific goals related to the module, and problem-solve potential barriers to success. For example, a patient working on the enhancing social support module might create a SMART goal (specific, measurable, achievable, realistic, and time-limited), to text a trusting neighbor twice in the coming week. Importantly, this process is client-directed, allowing for goals to be consistent with patient preferences and values at the time of the session. Clinicians utilize MI to help patients articulate the importance and confidence of their goals, which strengthens patients’ commitment and self-efficacy in meeting their goals. Finally, the clinician summarizes the session and asks the patient if there is anything to change or add to the summary to facilitate a shared understanding of the session and of the patient’s personalized goals. In keeping with the patient-centered principles of empowerment and flexibility, the patients choose whether to schedule another session (rather than being expected to attend a circumscribed number of sessions).

A randomized clinical trial (RCT) and earlier open pilot trial provide support for RISE’s efficacy in improving the psychosocial health of women VHA patients who experience past-year IPV [30,47]. The RCT compared RISE to an advocacy-based enhanced VHA care as usual intervention and found that RISE participants demonstrated significantly higher increases in empowerment (*d* = 3.46) and self-efficacy (*d* = 1.09) compared to the enhanced VHA care as usual participants [30]. In these research evaluations, we learned that RISE is acceptable to both patients and clinicians, as well as effective at enhancing psychosocial well-being among women VHA patients. However, it was unknown whether (a) the clinical benefits of RISE observed in the context of an RCT would transfer into routine VHA care, (b) whether RISE would be helpful for patients with other gender identities, and (c) whether patient outcomes differ by the type of IPV patients experience.

In this manuscript, we briefly describe VHA’s initial approach to implement RISE and we use program evaluation data from a cohort of patients who completed RISE at VHA hospitals to examine preliminary clinical effectiveness outcomes (i.e., psychosocial health and treatment satisfaction) from the pilot roll-out of RISE. We expected that patients who completed the RISE intervention would exhibit significant improvements on measures of general self-efficacy, depressive symptoms, and valued living from pretreatment to posttreatment. We also expected that patients would report high levels of satisfaction with the intervention. Although the pilot roll-out was originally intended for use with women, clinicians also administered RISE to men in response to referrals and clinical demand. Thus, as a secondary aim, we explore whether there are gender differences in treatment response and treatment satisfaction. Finally, we explored whether outcomes differed based on the types of IPV patients experienced. Modifications to RISE and its implementation characteristics are also discussed.

## 2. Materials and Methods

### 2.1. RISE Training Program Overview

#### 2.1.1. Training Sites and Participating Clinicians

As part of the IPVAP’s mission to assist Veterans who experience IPV, the IPVAP initiated a pilot implementation of RISE in Fiscal Year 20. They invited IPVAP Coordinators who were licensed clinicians at five VHA hospitals nationally that had established screening and referral pathways at their facilities to submit applications to become early adopters of RISE. Five individuals submitted applications that included documentation of local leadership support to implement RISE at their respective VHA hospitals, as such support is critical to successfully introducing new innovations in the healthcare setting [54,55]. All five applicants were selected. An additional site was added, for a total of six licensed clinicians. All sites participated voluntarily in the training program. One site participated in the training but dropped out without treating patients with RISE; this site is not included in the analyses. The training expectation was that all RISE-trained clinicians would begin using the intervention with women patients experiencing IPV within two weeks of completing the training and they would collect and submit clinical program evaluation metrics (described below).

#### 2.1.2. Training Process and Content

Clinicians participated in a pre-training orientation with leadership from the IPVAP to clarify expectations and pre-implementation tasks (e.g., raising awareness among other clinicians for RISE referrals). Clinicians received the RISE clinician manual and a patient handout manual and completed a self-study of the materials before the training. The clinician manual included information on the development of RISE and theoretical and empirical support, an overview of RISE’s underlying theory and treatment strategies, explanations of session structure, and core elements of the intervention.

Training was completed virtually by two licensed psychologists who were involved in developing and evaluating RISE and who both have substantial experience with IPV, trauma-informed care, and the VHA patient population [30,47]. A 5 h training focused on theoretical models and principles underlying the RISE intervention (e.g., empowerment and trauma-informed care), the general structure of the intervention (i.e., variable-length, modular, flexible), use of basic MI strategies (e.g., reflective listening), and role-plays for its use with Veterans experiencing IPV. The second half of the training focused on how to administer RISE, including safety check-ins and discussing general self-efficacy each session, describing each of the skills-focused modules and considerations for tailoring the modules, and goal-setting to the patients’ circumstances and preferences. The training concluded with considerations for maximizing safety, documentation, and mandated reporting issues. Following the training, clinicians received instructions for program evaluation data collection (e.g., timing of assessments, use of the IPVAP program evaluation portal to submit de-identified patient information and outcomes).

After each RISE session, clinicians were encouraged to complete a brief fidelity checklist to monitor their adherence to core elements of RISE. Clinicians attended bi-weekly virtual group clinical consultation with the RISE trainers for nine months while they implemented RISE. Clinicians graduated from the training program if they met the following criteria: (1) attended the full RISE training, (2) completed RISE cases with patients experiencing IPV (ongoing or within the past year) with an emphasis on fidelity to the treatment, (3) participated in at least 75% of consultation sessions, and (4) submitted program evaluation metrics. After meeting these requirements clinicians could continue administering RISE at their sites without participating in ongoing clinical consultation and program evaluation. RISE trainers and IPVAP leaders remained available for ad hoc clinical consultation or implementation support.

#### 2.1.3. Adaptations to RISE during the Pilot Implementation Initiative

Two noteworthy extensions of the RISE implementation protocol were enacted based on clinician request and necessity. First, clinicians desired to administer the intervention with men and non-binary-identifying patients. Second, clinicians efficiently switched to telemedicine at the start of the COVID-19 pandemic. Clinicians had experience delivering RISE via telemedicine; however, for some, this was the first time delivering IPV care using this modality. These adaptations were discussed during consultation sessions, with an eye for maximizing patient safety and privacy, and maintaining fidelity to the treatment. Accordingly, several consultation sessions were expanded to 90-min to enable additional time to share best practices, problem-solve barriers, and provide support to one another.

### 2.2. Program Evaluation Procedures

Clinicians completed summary forms for each RISE patient which included demographic information and types of IPV experienced (assessed during routine clinical practice), as well as the number of sessions attended, modules completed, and summary scores on program evaluation measures completed at the beginning and end of RISE. De-identified summaries were submitted into a RISE program evaluation portal housed on a secure VA SharePoint website. The Research and Development Committee of the VA Boston Institutional Review Board (IRB) reviewed the purpose and procedures and determined this program evaluation to be non-research and therefore exempt from further IRB review.

### 2.3. Program Evaluation Measures

General self-efficacy is the primary outcome for RISE and was measured with the 10-item General Self-Efficacy Scale [56] (GSES), which assesses optimistic self-beliefs to cope with difficult stressors in life [57]. Clinicians administer the GSES at the beginning of RISE (pretreatment assessment) and at each subsequent session, with the GSES from the last session considered the posttreatment assessment for analyses. Respondents rate statements such as “I am confident that I could deal efficiently with unexpected events” on a 4-point Likert scale from 1 (*not at all true*) to 4 (*exactly true*). Items are summed (range: 10–40), with higher scores indicating greater self-efficacy. In past research, the GSES has demonstrated good internal reliability (αs range from 0.76 to 0.90) and criterion-related validity (e.g., positively correlated with hope for success [ *r* = 0.43]) [57].

Depressive symptoms were measured using the 7-item depressive subscale of the Depression, Anxiety, Stress Scale-21 [58] (DASS-21). Patients rate the past-week frequency of experiencing depressive symptoms using a 4-point scale from 0 (did not apply to me) to 3 (applied to me very much, or most of the time). Items are summed and multiplied by two for a total score (range: 0–42), with higher scores indicating greater symptoms. In addition to overall symptoms, we examined the presence of probable depression diagnoses (score ≥ 14) at pretreatment and posttreatment [59]. In prior research, the DASS-21 depressive subscale has demonstrated good internal consistency reliability (α = 0.82) and construct validity (e.g., negatively correlated with positive affect [r = −0.48]) [60].

Valued living was measured using the Valued Living Questionnaire [61] (VLQ), which assesses the alignment of one’s daily life with one’s values. This measure uses a 10-point scale to assess (a) the importance of 10 valued domains (e.g., self-care, friendship, work), and (b) the extent to which the individual has lived in accordance with those values in the past week. Scales are multiplied for a composite score (10–40), with higher scores reflecting greater valued living. In prior research, the VLQ has shown adequate internal consistency reliability (αs range from 0.64 to 0.77) and construct validity (e.g., positive association with vitality [r = 0.27]) [62].

*Treatment satisfaction* was assessed using the 8-item Client Satisfaction Questionnaire [63] (CSQ-8). Items are scored on a 4-point Likert scale from 1 (*low satisfaction*) to 4 (*high satisfaction*). Items are summed for a total score (range: 8–32), with higher scores indicating greater satisfaction. In prior research, the CSQ-8 has demonstrated excellent internal consistency reliability (α = 0.93) and construct validity (e.g., greater satisfaction is associated with lower mental health symptoms [r = −0.40]) [64,65].

### 2.4. Analyses

We computed descriptive statistics on patient sociodemographic characteristics to describe the patients and their participation in the intervention (e.g., number of sessions attended). Next, we computed descriptive statistics and paired sample *t*-tests to examine significance of change from pretreatment to posttreatment on self-efficacy, depressive symptoms, and valued living. Patients who had the majority of program evaluation measures were included in these analyses (*n*’s range from 39–45). Cohen’s *d* effect sizes were calculated, with interpretations of 0.2 = small effect, 0.5 = medium effect, and 0.8 = large effect [66]. McNemar’s chi-square test was used to examine change in the proportion of patients who had probable depression at pretreatment and posttreatment. Treatment satisfaction was examined descriptively focusing on mean satisfaction scores.

We additionally explored whether there were differences in treatment impact or satisfaction by gender. For the pre-to-post measures, we created difference scores for each outcome (posttreatment scores—pretreatment scores). We conducted a series of one-way between-groups analyses of variance (ANOVAs) using the difference score as the outcome and gender (women/men) as the grouping variable. For treatment satisfaction, we also conducted a one-way between groups ANOVA using the CSQ-8 total score as the outcome and gender (women/men) as the grouping variable.

Finally, given that different types of interpersonal traumas can differentially impact health outcomes [67,68] and may therefore impact response to treatment, we explored whether there were differences in outcomes by IPV type. We conducted a series of one-way between-groups ANOVAs using the differences scores as the outcomes and the types of IPV experienced as a three-level grouping variable consisting of: (1) psychological IPV only, (2) psychological and physical IPV only, and (3) psychological, physical and sexual IPV. We also conducted a one-way between groups ANOVA for treatment satisfaction using the CSQ-8 total score as the outcome and IPV type as the grouping variable.

## 3. Results

### 3.1. Patient Characteristics and Engagement in RISE

There were 45 patients who received RISE and completed the majority of program evaluation measures for the pilot. Table 2 displays patient sociodemographic and IPV characteristics. The sample predominately identified as women (84.4%) and approximately 40% of the patients identified as a racial/ethnic minority. In the past year, all patients reported experiencing psychological IPV (*n =* 45, 100%), two-thirds reported experiencing physical IPV (*n =* 30, 66.7%), and roughly one-fifth of the sample experienced sexual IPV (*n* = 10, 22.2%).

The average number of RISE sessions received was six (*M* = 5.60, *SD* = 2.27). The percentage of patients selecting each module were as follows: Improving Coping and Self-Care (84.4%; *n* = 38), Education on Health Effects of IPV and Warning Signs (75.6%; *n* = 34), Making Difficult Decisions (73.3%; *n* = 33), Connecting with Resources and Moving Forward (66.7%; *n* = 30), Safety Planning (53.3%; *n* = 24), and Enhancing Social Support (42.2%; *n* = 19).

### 3.2. Patient Clinical Outcomes

Paired-sample *t*-tests were conducted on the three psychosocial health measures to evaluate the clinical impact of the RISE intervention on patient health. There were significant main effects for time on all within-subjects comparisons (two-tailed); see Table 3 for descriptive statistics and *t*-test values related to these results. Specifically, there was a statistically significant increase in self-efficacy and valued living scores from pretreatment to posttreatment (Cohen’s *d* = 0.97 and 0.51, respectively) as well as a statistically significant decrease in depressive symptoms from pretreatment to posttreatment (Cohen’s *d* = 1.09). In addition, of the 39 patients with pretreatment and posttreatment data on the DASS-D, 74.4% of the sample *(n* = 29) had probable depression at pretreatment whereas only 33.3% of the sample (*n* = 13) had probable depression at posttreatment (McNemar test *p* < 0.001).

Satisfaction scores on the CSQ-8 were calculated at post-treatment (*n* = 30). Satisfaction ratings were high, with an average CSQ-8 score of 30.47 (SD = 1.98). Considering the upper most score of the CSQ-8 is 32, these scores reflect overall satisfaction as excellent across the sample.

### 3.3. Additional Analyses to Explore Outcomes by Gender and IPV Types

First, we conducted a series of one-way between-groups ANOVAs to examine whether there was a difference in clinical outcomes by gender. As summarized in Table 4, there were no statically significant differences by gender on any of the psychosocial health outcomes or on treatment satisfaction. Although caution should be used in interpreting these findings given the very small sample for men, these findings suggest there were no gender differences on psychosocial health outcomes or treatment satisfaction in the current sample.

Next, we conducted a series of one-way between-groups ANOVAs to examine whether there was a difference in clinical outcomes by IPV type. As displayed in Table 5, there were no significant differences by IPV type on psychosocial health impacts or treatment satisfaction.

## 4. Discussion

There is a critical need for IPV interventions in healthcare settings, particularly in those that serve Veterans as they experience substantial risk for recent IPV and associated health problems, yet few specialized interventions exist for Veteran-specific contexts [6]. This manuscript describes a pilot rollout of RISE in the US VHA, the nation’s largest integrated healthcare system, and presents clinical outcomes from patients who received the intervention across five VHA hospitals. This was an observational pre-post program evaluation of patients who received RISE and completed program evaluation measures, and thus, readers should consider the potential causal effect of RISE with this design issue in mind. Patients who received RISE reported significant improvements on all of the psychosocial health measures from pretreatment to posttreatment and reported high levels of satisfaction with the RISE intervention. Findings provide preliminary support for the effectiveness and the acceptability of RISE with men, as results were similar across gender. Furthermore, outcomes did not differ based on the types of IPV experienced, suggesting RISE may be similarly effective and acceptable across IPV types. Taken together, these findings support the feasibility of implementing RISE in VHA while also demonstrating the preliminary effectiveness of RISE in this routine care context.

Not only were clinical symptom changes from pretreatment to posttreatment statistically significant, but also the magnitude of improvements were medium to large, consistent with RCT findings [30]. Patients’ average general self-efficacy scores at pretreatment were slightly lower than typically seen in IPV intervention studies [69], but at posttreatment, most patients’ general self-efficacy scores were above national US norms on this measure [70]. This is promising as several prior IPV intervention studies have demonstrated limited improvements in self-efficacy [69,71]. Additionally, patients’ average score was in the moderate range of depression at pretreatment and most of the patients reported no depressive symptoms or mild symptoms at posttreatment [59]. Thus, RISE provided in routine care helps reduce general psychological distress and feelings of overwhelm that are common among individuals experiencing IPV [72,73]. Reductions in distress may in turn enhance survivors’ use of internal (e.g., self-care) and external resources (e.g., social support) to effectively cope with and recover from the far-reaching impacts of IPV and increase safety [74,75,76,77]. Similarly, increases in valued living is important for patients impacted by IPV. A core aspect of IPV is disempowerment, and that process may be compounded by the experience of stress in marginalized populations who experience unique barriers to care [78,79]. Supporting patients experiencing IPV from within an empowerment framework to connect with their values may be particularly impactful. For example, working with a black transgender patient to reengage with her church might be one values-based goal to pursue during RISE.

Beyond traditional clinical outcomes, an important aspect of implementing evidence-based interventions into routine care is the acceptability of the treatment from the patient perspective [80]. The patients in this program evaluation reported high levels of satisfaction with RISE, extending prior research findings demonstrating the acceptability of RISE from the patient and provider perspectives [30,81]. Moreover, this program evaluation pilot included men, and although the content of the intervention manual and handouts were originally intended for women, men reported high levels of overall satisfaction with RISE suggesting that clinicians were able to effectively tailor the intervention to their needs. That outcomes did not differ based on the combination of types of IPV experienced suggests that RISE is helpful for patients with various types of IPV experiences.

In response to clinician needs during the pilot implementation, the following modifications were made to the RISE intervention and implementation characteristics. First, the program materials were edited to be more gender neutral in language, visual imagery, and examples provided to ensure they were relatable to a diverse array of individuals. Additionally, modifications included efforts to create a more affirming and inclusive protocol, aligned with the principles of trauma-informed care for LGBTQ+ persons [78]. Second, we increased the flexibility regarding how many sessions an individual could attend, to allow for greater individualization of needs and ensure all facility-level requirements (e.g., comprehensive suicide risk assessments) could be accomplished while still getting a full dose of RISE. Moreover, clinicians felt some patients would benefit from additional sessions to consolidate skills and plan for potential setbacks, consistent with IPV survivor preferences for IPV interventions [81]. This attention to increased flexibility and collaboration paralleled additional changes made to deliver the protocol during the COVID-19 pandemic, including how clinicians and patients must develop ‘work-arounds’ during COVID-19 [82]. For example, RISE clinicians rapidly shifted to facilitating ‘counseling in cars’ at the onset of the pandemic, as often, the only time patients had privacy (e.g., from abusive partners) was when they were parked in their car in a safe location. Third, a module to address the unique experiences of sexual violence across the lifespan was developed after the pilot based on feedback from RISE clinicians and IPVAP leadership. This facilitates psychoeducation and safety planning for more recent experiences of sexual coercion or assault while also making connections between the sexual assault histories and current health needs. This reflects the fact that a notable proportion of Veterans report histories of sexual violence (i.e., child sexual abuse, unwanted sexual experiences during military service) in combination with their recent sexual and non-sexual IPV experiences [19,83,84,85].

Formative program evaluation is ongoing in the VHA to examine the revised RISE intervention and implementation characteristics in a subsequent cohort of pilot sites. In this second pilot, the IPVAP engaged and trained clinicians at multiple sites with identified RISE clinical champions to increase the reach of RISE and encourage team-based implementation, as has been done in other successful implementation efforts in the VHA [26]. These refinements to RISE and iterative revisions to IPVAP’s implementation strategies align with best practices for developing implementation strategies [86] and are informing a larger-scale plan to scale-up and spread RISE in the VHA.

### Limitations

The program evaluation data has limitations. First and foremost, the basic observational pre- and post-test design imparts limitations on the interpretation of findings. The lack of a control group means we cannot determine whether improvements in health were truly a reflection of the specific treatment components of RISE or whether they could have been due to other nonspecific factors (e.g., passing of time or behavioral activation associated with participating in a structured intervention). Additionally, it is possible that there were patient-selection bias present in this evaluation. RISE may have been offered to patients that clinicians thought were most likely to benefit from the treatment. Moreover, clinicians predominantly submitted treatment summaries for patients who completed at least a few sessions of RISE and therefore we are unable to compare those who did and did not complete RISE on patient characteristics or outcomes. This sample was comprised largely of heterosexual, cisgender women. Although findings suggest RISE’s potential utility for men, a larger evaluation that includes a more diverse and representative sample of VHA patients is needed. In particular, VHA patients identify as sexual and gender minorities at elevated rates compared to the general population [87] and they report substantial IPV exposure and associated mental and physical health consequences [88,89].

## 5. Conclusions

Results from the pilot implementation of RISE provide support for the intervention’s effectiveness and acceptability in routine clinical care in the VHA. These findings include no research-specific procedures, but rather reflect outcomes in a real-world setting and therefore suggest program generalization. Findings also provide early support for applying the RISE intervention with men and reinforce that RISE can address the needs of patients who experience various types of IPV. These findings are informing the scale-up of RISE in the VHA and may be helpful to other healthcare settings that desire to implement psychosocial counseling interventions for patients who experience IPV. In particular, IPV is a common social determinant of health among Veterans and there is a strong need for evidence-based interventions to be implemented in Veteran-specific settings [90].

## Figures and Tables

**Table 1:** Descriptions of the Modules Patients Select from during a RISE Session.

Module Title	Brief Description
Safety Planning	Ways to increase your safety, and that of any children and pets, in different situations, such as in an argument or if you are thinking about ending the relationship through worksheets and other user-friendly safety planning tools.
The Health Effects and Warning Signs of IPV	Understanding the effects of trauma and IPV on different aspects of your life (for example, your physical, mental, and social health, and the well-being of your children). Learn about warning signs of IPV, including red flags in relationships, as well as differences between aggressive and assertive behavior.
Improving Coping and Self-Care	Learning about the importance of self-care and practicing ways to relax when you are stressed, as well as practicing basic coping skills and self-care strategies to manage difficult emotions and situations.
Enhancing Social Support	Learning ways to increase social support as well as evaluating the support system that’s already in place. Learning and practicing how to approach friends or family to ask for support and how to know what healthy, trustworthy support looks like in various types of relationships.
Making Difficult Decisions	Learning skills to help you think about your options and making difficult decisions. Utilize worksheets to weigh the pros and cons of important decisions, especially if you are thinking about making a change in your relationship or in another important area of your life.
Resources and Moving Forward	Educating and linking you to resources available in the community for a variety of topics (such as housing, employment, legal aid, and restraining orders). Be reminded of the things you have accomplished during RISE and plan ahead for life’s ups and downs by identifying red flags to watch out for and various ways to cope.

Note. Sexual Violence Over the Lifespan module was added as a result of stakeholder feedback from the pilot implementation project. This module was added *after* the project was complete and is now part of the RISE treatment, as noted in the Discussion.

**Table 2:** Characteristics of the Sample (*N* = 45).

	Number (*n*) *^a^*	Percent
Race/Ethnicity		
Black	10	22.2
Asian	3	6.7
White	27	60
Unknown/Omitted	5	11.1
Hispanic	4	8.9
Gender Identity		
Woman Cisgender	37	82.2
Woman Transgender	1	2.2
Male Cisgender	7	15.6
Sexual Orientation		
Heterosexual/Straight	40	88.9
Lesbian/Gay	2	4.4
Pansexual	1	2.2
Unknown/Omitted	2	4.4
Types of IPV Present		
Physical	30	66.7
Sexual	10	22.2
Psychological	45	100
Stalking	11	24.4
Relationship Status		
Married	12	26.7
Divorced	5	11.1
Separated	9	20.0
Never Married	2	4.4
Currently Dating	8	17.8
Single—Not Dating	10	22.7
Relationship to Person Using Violence		
Current Partner	16	35.6
Former Partner	28	62.2
Unknown/Omitted	1	0.02

Note. *^a^* Not all *n*’s add up to 45 due to occasional missing data and some categories are not mutually exclusive.

**Table 3:** Descriptive statistics and changes in psychosocial health outcomes from pretreatment to posttreatment.

Variable	Pretreatment	Posttreatment	Lower	Upper	Range	*t*	*d*
GSES	26.56 (5.96)	32.24 (5.58)	3.92	7.46	10–40	6.48 **	0.97
DASS-D	19.38 (10.93)	9.33 (8.43)	7.05	13.05	0–42	6.78 **	1.09
VLQ	34.36 (26.62)	38.42 (28.58)	0.95	7.16	10–100	2.68 *	0.51

Note. N’s do not all equal 45 due to occasional missing data. Range = Range of possible scores on the measure. GSES = General Self-Efficacy total score. DASS-D = Depressive symptoms total score. VLQ = Valued living total score. Lower Upper = 95% confidence interval of the difference. * *p <* 0.05; ** *p <* 0.001.

**Table 4:** Means, Standard Deviations, and One-Way Analyses of Variance for the Effects of Gender on Psychosocial Health and Satisfaction with Treatment.

	Women		Men			
Variable	*M*	SD	*M*	SD	*F*	*p*
GSES	5.71	5.36	5.57	8.81	0.003	0.96
DASS-D	−9.52	8.14	−13.00	14.68	0.71	0.40
VLQ	4.01	8.21	4.23	7.88	0.004	0.95
CSQ-8	30.54	2.04	30.17	1.83	0.17	0.69

Note. Descriptive statistics for psychosocial health variables reflect change scores during treatment while treatment satisfaction is the total score reported at posttreatment. GSES = General self-Efficacy difference score. DASS-D = Depressive symptoms difference score. VLQ = Valued living difference score. CSQ-8 = Treatment satisfaction total score at posttreatment.

**Table 5:** Means, Standard Deviations, and One-Way Analyses of Variance for the Effects of IPV Type on Psychosocial Health Outcomes and Satisfaction with Treatment.

	Psych Only		Psych & Phys		Psych, Phys, & Sexual			
Variable	*M*	SD	*M*	SD	*M*	SD	*F*	*p*
GSES	5.62	4.70	6.32	6.39	4.13	7.10	0.38	0.68
DASS-D	−9.17	8.11	−11.16	9.44	−13.33	8.73	0.46	0.64
VLQ	7.34	9.66	0.67	6.45	6.77	5.02	2.31	0.12
CSQ-8	31.20	1.03	30.47	1.77	31.33	1.15	0.92	0.41

Note. Descriptive statistics for psychosocial health variables reflect change scores during treatment while treatment satisfaction is the total score reported at posttreatment. Psych Only = Psychological IPV only. Psych & Phys = Psychological and Physical IPV only. Psych, Phys, & Sexual = Psychological, Physical & Sexual IPV. GSES = General Self-Efficacy difference score. DASS-D = Depressive symptoms difference score. VLQ = Valued living difference score. CSQ-8 = Treatment satisfaction total score at posttreatment.

## Data Availability

The dataset generated during and/or analyzed during the current study represent program evaluation that is not publicly available.
